# Dual Attention-Based 3D U-Net Liver Segmentation Algorithm on CT Images

**DOI:** 10.3390/bioengineering11070737

**Published:** 2024-07-20

**Authors:** Benyue Zhang, Shi Qiu, Ting Liang

**Affiliations:** 1 Key Laboratory of Spectral Imaging Technology CAS, Xi’an Institute of Optics and Precision Mechanics, Chinese Academy of Sciences, Xi’an 710119, China; zhangbenyue22@mails.ucas.ac.cn; 2 School of Optoelectronics, University of Chinese Academy of Sciences, Beijing 100408, China; 3 Department of Radiology, The First Affiliated Hospital of Xi’an Jiaotong University, Xi’an 710119, China

**Keywords:** dual attention mechanisms, residual connection, 3D U-Net, CT, liver segmentation

## Abstract

The liver is a vital organ in the human body, and CT images can intuitively display its morphology. Physicians rely on liver CT images to observe its anatomical structure and areas of pathology, providing evidence for clinical diagnosis and treatment planning. To assist physicians in making accurate judgments, artificial intelligence techniques are adopted. Addressing the limitations of existing methods in liver CT image segmentation, such as weak contextual analysis and semantic information loss, we propose a novel Dual Attention-Based 3D U-Net liver segmentation algorithm on CT images. The innovations of our approach are summarized as follows: (1) We improve the 3D U-Net network by introducing residual connections to better capture multi-scale information and alleviate semantic information loss. (2) We propose the DA-Block encoder structure to enhance feature extraction capability. (3) We introduce the CBAM module into skip connections to optimize feature transmission in the encoder, reducing semantic gaps and achieving accurate liver segmentation. To validate the effectiveness of the algorithm, experiments were conducted on the LiTS dataset. The results showed that the Dice coefficient and HD95 index for liver images were 92.56% and 28.09 mm, respectively, representing an improvement of 0.84% and a reduction of 2.45 mm compared to 3D Res-UNet.

## 1. Introduction

Liver diseases account for two million deaths annually and are responsible for 4% of all deaths, posing a significant impact on human health and socio-economic factors [1,2]. Medical imaging plays a crucial role in the diagnosis and treatment of liver diseases, and computed tomography (CT) is the primary modality for obtaining liver images. Liver CT image segmentation allows for the quantitative assessment of liver morphology, size, density, and other indicators, aiding physicians in disease evaluation and clinical research, such as liver tumors and cirrhosis. However, traditional manual methods for liver image analysis are time-consuming, and the accuracy of analysis is positively correlated with the experience of the physician, leading to variability in diagnoses among different physicians. Therefore, there is a need to establish unified standards and utilize artificial intelligence methods to assist physicians in accurate diagnosis.

Liver automatic segmentation aims to use computer vision and deep learning techniques to automatically identify and segment liver regions in medical images. Compared to traditional manual segmentation methods, automatic segmentation offers advantages such as faster processing speed, higher accuracy, and better repeatability, which are essential for improving the efficiency and accuracy of medical image analysis.

Deep learning algorithms have become the mainstream approach for liver automatic segmentation. Among them, convolutional neural networks (CNNs) are one of the most commonly used deep learning models. In particular, fully convolutional networks (FCNs) [3] and their improved versions, such as U-Net [4], have achieved significant success in liver automatic segmentation tasks. These methods, through end-to-end training, can directly learn liver segmentation results from raw images, demonstrating high accuracy and robustness. Meng et al. [5] proposed a two-stage liver segmentation algorithm based on CNNs, leveraging shallow feature spatial information to enhance liver segmentation capability. Xi et al. [6] introduced cascade U-ResNets, connecting two U-Nets with ResNet encoders and inter-network skip connections, demonstrating superior liver segmentation performance compared to single models. Zhang et al. [7] integrated auto-context elements into a U-Net-like architecture and introduced a patch-based strategy and weighted sampling procedure, testing on a dataset of 20 patients with results surpassing the benchmark method for liver tissue classification. Li et al. [8] proposed the RDCTrans U-Net, which combined the U-Net architecture with a hybrid variable structure with a backbone based on ResNeXt50 and introduced a Transformer in the downsampling process. This model achieved a Dice coefficient of 93.38%. Wei et al. [9], based on the High-Resolution Network (HRNet), proposed a high-resolution Swin Transformer Network (HRSTNet) using Transformer blocks instead of traditional convolution modules, achieving superior results in precise liver segmentation. Gao et al. [10] used V-Net to segment the ROI and further refined the segmentation with a hybrid LSM, significantly improving the sensitivity, precision, and Dice coefficient of liver segmentation. Xing et al. [11] integrated a diffusion model into a U-shaped architecture and introduced a Step-Uncertainty-based Fusion (SUF) module, showing that Diff-UNet significantly outperformed other state-of-the-art methods. Bogoi et al. [12] compared the lightweight model UNeXt with U-Net, utilizing soft dice loss and unified focal loss, replacing the traditional ReLU activation function with the Funnel activation function, greatly reducing the computational burden of similar models. Liu et al. [13] introduced a Multi-scale Feature Extraction and Enhancement U-Net (mfeeU-Net), combining Res2Net blocks, Squeeze-and-Excitation (SE) blocks, and Edge Attention (EA) blocks, effectively addressing issues of liver region discontinuity and fuzzy liver boundaries. Kushnure et al. [14] introduced a deep learning-based lightweight multi-level multiscale approach with deep residual learning called LiM-Net, integrating channel-wise attention (PARCA) block into the Unet++ framework for refinement, achieving high segmentation accuracy across major liver datasets. Luan et al. [15] proposed S-Net, introducing attention mechanisms between the contraction path and the expansion path and using long-hop connections to merge semantic information, enhancing the recognition of tumors in CT images. Pettit et al. [16], based on nnU-Net, used CT images for accurate liver volume estimation, achieving a high accuracy rate of 97.5% in identifying liver boundaries. Almotairi et al. [17] achieved good liver segmentation results by combining SegNet with semantic pixel-wise classification of road scenes technology. Liu et al. [18] proposed the dense feature selection U-Net (DFS U-Net) and improved the loss function, demonstrating the model’s efficiency, high effectiveness, and robustness in clinical applications. Wardhana et al. [19] extensively studied the parameters of a 2.5D model-based CNN for automatic liver segmentation, proving that multiple stacked layers perform better than a single-layer network. Lei et al. [20] proposed a deformable encoder-decoder network (DefED-Net) using deformable convolutions and a Ladder Atrous Spatial Pyramid Pooling (Ladder-ASPP) module to enhance representations and learning contextual information, achieving higher accuracy in liver segmentation compared to SOTA. Mourya et al. [21] introduced a dilated deep residual network (DDRN), where three parallel DDRNs and a fourth DDRN cascaded together achieved outstanding results in an online evaluation system. Tian et al. [22], based on the Res-UNet architecture, proposed a 2.5D class-aware deep neural network with spatial adaptation for automatic functional region annotation of the liver, adaptively selecting neighboring CT slices as inputs and generating masks corresponding to the central slice, achieving a high average Dice score and low computational cost. Hong et al. [23] proposed an unsupervised domain adaptation framework for cross-modality liver segmentation via joint adversarial learning and self-learning, achieving high Dice scores on public datasets. Tan et al. [24] proposed an automatic liver segmentation framework based on three-dimensional (3D) convolutional neural networks with a hybrid loss function suitable for training on small datasets. Jeong et al. [25] introduced an automated liver segmentation and volume estimation method using deep learning, achieving high segmentation precision. Pandey et al. [26] harnessed the capabilities of the YOLOv8 model for approximate boundary box detection across modalities alongside the Segment Anything Model (SAM) and High Quality (HQ) SAM for fully automatic and precise segmentation. Lin et al. [27] explored the feasibility of using EfficientNetV2 as an encoder in combination with U-net. Experimental results indicated that employing EfficientNetV2 as an encoder together with U-net can improve the segmentation model’s Dice score. Cheng et al. [28] adapted SAM to medical image segmentation through more comprehensive prompts involving bounding boxes, points, and masks. SAM-Med2D demonstrated significantly superior performance and generalization capability compared to SAM.

The main challenges faced by the aforementioned algorithms are as follows:

(1) Limited network depth restricts the extraction and modeling ability of complex features.
(2) Lack of comprehensive global contextual analysis capability limits performance improvement.
(3) Loss of semantic features during the upsampling process results in decreased accuracy of the final prediction.

To address these challenges, this paper proposes an improved dual attention mechanism 3D U-Net network, introducing residual connections and CBAM and integrating DA-Block designed specifically for extracting image positional and channel features, effectively enhancing the accuracy of medical image segmentation. The innovations of this paper are summarized as follows:

(1) An improved 3D U-Net network with residual connections is introduced to increase network depth, better capture multiscale information, and alleviate semantic information loss.
(2) Integration of DA-Block into the encoder aims to enhance feature extraction capability and extract rich global contextual information.
(3) The introduction of CBAM modules in each skip connection optimizes the transfer of features in the encoder, reduces semantic gaps, aids the decoder in reconstructing feature maps, and achieves accurate liver segmentation.

Experiments conducted on the LiTS dataset demonstrated that the proposed method improved the performance of medical image segmentation. The rest of this paper is organized as follows: Section 2 provides a review of the related works of automatic liver image segmentation. Our proposed method is introduced in Section 3. Next, the detailed experiments and analyses are conducted in Section 4. Finally, Section 5 makes a conclusion of the whole work.

## 2. Related Works

Currently, methods for liver medical image segmentation can be categorized into manual segmentation, semi-automatic segmentation, and automatic segmentation. Manual segmentation relies on experts to manually annotate images based on their experience, which is not only time-consuming but also prone to issues like unclear liver edge features. Semi-automatic segmentation methods are relatively simple and fast, but the segmentation results depend on the initial parameter settings, leading to subjective variability in the algorithm. Automatic segmentation methods typically employ FCNs and have made significant advancements in liver medical image segmentation. This section provides a comprehensive analysis of various automatic methods employed for segmenting the liver from CT images.

### 2.1. U-Net Models

To address the issue of limited samples affecting the effectiveness of automatic segmentation, Ronneberger et al. [4] proposed U-Net, which can be trained with a small amount of data to generate precise segmentation results. Consequently, many medical image segmentation problems have been improved based on U-Net. Depending on the dimensionality of the network input, U-Net, and its modified versions can be categorized into liver segmentation methods with 2D input, contextual information input, and 3D input.

For liver segmentation methods with 2D input, Liu et al. [29] achieved higher-level semantic features by increasing the depth of U-Net. They refined the segmentation results using graph cuts to smooth the boundaries more effectively. Li et al. [30] proposed the bottleneck feature supervised U-Net, addressing the issue of semantic information loss during downsampling by incorporating dense and inception modules. This approach pays closer attention to liver boundaries, thereby improving segmentation accuracy. Wu et al. [31] introduced MSA-UNet, which combines low-level and high-level features and integrates a multiscale attention mechanism to achieve higher precision in liver segmentation. Yu et al. [32] introduced an attention mechanism in DResUNet, which utilizes deformable convolutions to fuse multiscale features in both spatial and channel dimensions. Jiang et al. [33] introduced Res-SE-Block and Multi-Attention block in RMAU-Net. The former alleviates gradient issues and enhances feature quality, while the latter captures multiscale information and constructs inter-feature relationships. They also proposed a hybrid loss function (focal and dice) to improve segmentation accuracy and network convergence speed.

For liver segmentation methods integrating contextual information input, since most liver image data are in 3D format, many “2.5D” networks have emerged to incorporate contextual information. Tian et al. [22] proposed a 2.5D class-aware deep neural network with spatial adaptation for automatic functional region annotation of the liver based on the Res-UNet architecture. This method adaptively selects adjacent CT slices as input and generates masks corresponding to the central slice, achieving high average Dice scores with low computational costs. Chen et al. [34] introduced Chanel-UNet, which utilizes spatial-channel convolutions to extract mapping relationships of spatial information between pixels in the feature maps, effectively optimizing the extraction of spatial information in spatial-channel convolutions. To increase the receptive field in three dimensions, Han et al. [35] proposed 2.5D Perpendicular-UNet, which allows customization of data augmentation, loss functions, and post-processing steps. Lv et al. [36] presented RIU-Net, which takes adjacent slices of liver CT images as input and incorporates residual blocks and InceptionV3 to extract more image features while reducing network parameter quantity.

For liver segmentation methods with 3D input: The 3D U-Net [37] is specifically designed to handle three-dimensional volumes. This network comprises two main components: an encoder and a decoder. The encoder extracts features from the input data using multiple convolutional layers, followed by max-pooling operations to reduce spatial dimensions and increase feature channel numbers. This iterative process captures intricate feature details. The decoder connects with the feature maps in the encoder through upsampling. Upsampling reduces feature channel numbers but increases spatial dimensions. This design enables the network to reconstruct input images at the original resolution while retaining high-level features learned by the encoder. Czipczer et al. [38] adopted the 3D U-Net as the basic structure and combined probability density function estimation and supervoxel segmentation, which is more suitable for liver segmentation with limited datasets. Chi et al. [39] proposed a multi-branch network, introducing an up-sampling branch for liver region recognition and a pyramid-like convolution structure for inner-liver feature extraction in Dense U-Net. They simplified the convolution operations in traditional 3D U-Net, improving segmentation accuracy and significantly reducing computational complexity. To address the issue of insufficient training data due to the difficulty in obtaining labeled data, He et al. [40] devised a semi-supervised optimization algorithm integrating 3D U-Net into the Generative Adversarial Networks (GANs) framework, yielding promising liver segmentation results.

### 2.2. The Use of Attention Mechanisms in Liver Segmentation

To extract more feature information, some liver segmentation studies focus on attention mechanisms, which fuse high-level features with low-level features containing image details to improve segmentation accuracy. Chen et al. [41] proposed DRAUNet, which introduces deep residual blocks (DR blocks) and dual-effect attention modules (DAM) to fuse feature channel correlations and spatial feature map information. This model performed exceptionally well on various datasets. Based on attention-based feature fusion methods, the low-level features generally extract minimal semantic information. To effectively embed more semantic information into the low-level features, Chen et al. [42] designed an attention-based feature fusion method to merge features from different levels. Additionally, to compensate for information loss during the upsampling process, a dense upsampling convolution and residual convolution structure were proposed, ultimately achieving higher segmentation accuracy. Guo et al. [43] proposed an innovative lightweight network, SA-UNet, which incorporates a spatial attention module to infer attention maps along the spatial dimension and perform adaptive feature refinement, effectively enhancing the accuracy of feature extraction. Additionally, by utilizing structured dropout convolutional blocks to prevent overfitting, SA-UNet achieves outstanding segmentation performance even without a large number of annotated samples.

## 3. Methodology

3D U-Net [37] demonstrates strong performance in processing three-dimensional data, but it still exhibits some inherent shortcomings: (1) The 3D U-Net has relatively few network layers, which limits its ability to model complex features; (2) During the upsampling process, 3D U-Net tends to lose a significant amount of semantic features, potentially decreasing the accuracy of the final prediction results; (3) The global context analysis capabilities of 3D U-Net remain to be enhanced. Given these limitations, 3D U-Net faces challenges in meeting increasingly complex diagnostic demands, necessitating improvements to its network architecture to enhance its representation of complex features and reduce semantic information loss.

**Figure 1:**
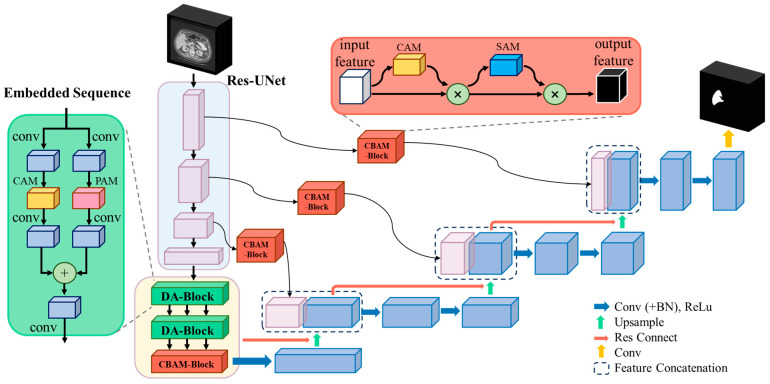
The architecture of improved dual attention mechanism-based 3D U-Net. After inputting liver CT images, the images undergo four rounds of downsampling and are then fed into embedding layers containing DA-Blocks and CBAM. During the downsampling process, CBAM is utilized for feature extraction, which is then fused with the decoder via skip connections. After four corresponding upsampling layers, the channels are restored to the same resolution as the input image, resulting in the final liver image segmentation outcome.

The method proposed in this paper builds upon the 3D U-Net framework, incorporating residual connections in each encoder and decoder module and integrating the Dual Attention Block (DA-Block) and Convolutional Block Attention Module (CBAM) at every layer connection of the encoder and decoder to enhance performance. Figure 1 illustrates the modified 3D U-Net architecture and procedural steps. The DA-Block extracts features based on position and channels, while the CBAM focuses on spatial and channel-based feature extraction. On the one hand, the DA-Block in the encoder refines the input feature maps, obtaining more accurate and detailed global features. On the other hand, the CBAM in the skip connections optimizes the features transferred from the encoder, assisting the decoder in reconstructing more accurate feature maps, thereby improving the model’s segmentation capability.

### 3.1. 3D Res-UNet

**Figure 2:**
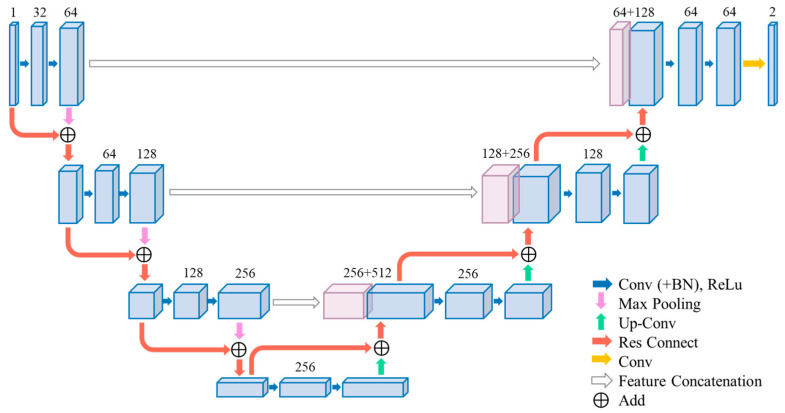
Structure of 3D Res-UNet. Residual connections are added in each encoding block and decoding block.

The specific structure of the 3D Res-UNet is shown in Figure 2. Similar to the architecture of 3D U-Net, 3D Res-UNet also consists of encoders and decoders, with residual connections [44,45] incorporated into each encoder and decoder module, as indicated by the red arrows in Figure 1 and Figure 2.

(1) The encoder module utilizes convolution and pooling operations to downsample the input, progressively reducing the image size and feature count to extract low-level features.
(2) The decoder module employs transpose convolution operations to upsample the input, gradually increasing the image size and feature count, and performs feature fusion to generate high-level features.
(3) The residual connections are responsible for connecting features with the same resolution during the downsampling and upsampling processes, aiding the network in better capturing multiscale information, alleviating semantic information loss issues, and enhancing image segmentation performance.

Residual connections are implemented as follows:

(1) $$y=F\left(x,\left\{w_{i}\right\}\right)+H\left(x\right)$$

where *x* represents the input and *y* represents the output. *F* consists of two convolution operations and one max-pooling operation or upsampling operation. *H* denotes the same mapping or convolution operation, ensuring that the feature dimensions of the input are the same as those of *F*.

### 3.2. Dual Attention Block

The Dual Attention Block (DA-Block) [46,47], serving as a feature extraction module, integrates position-based and channel-based feature extraction components, enabling the model to capture feature information from multiple perspectives. By extracting features from multiple perspectives, more accurate and detailed feature information can be obtained, thus, enhancing the model’s performance in segmentation tasks. In this study, DA-Blocks are incorporated into the last layer of the encoder to enhance the segmentation performance of the model.

**Figure 3:**
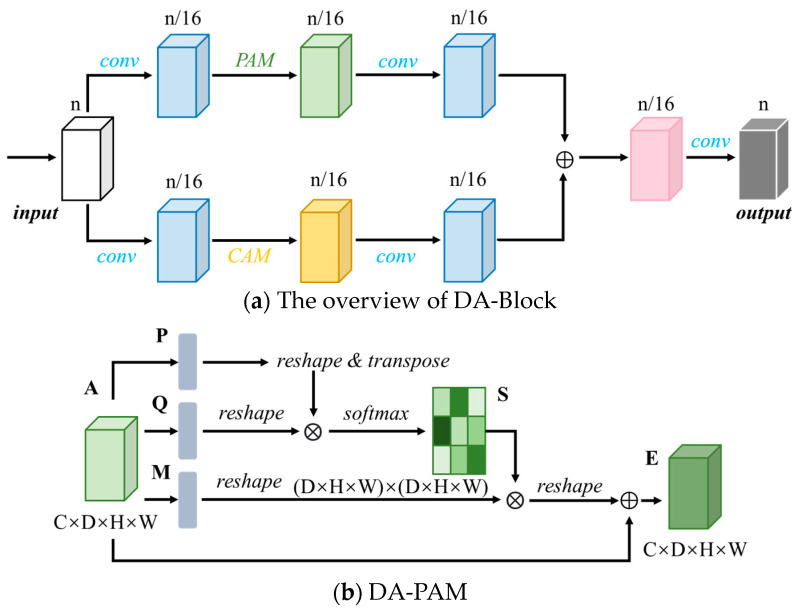

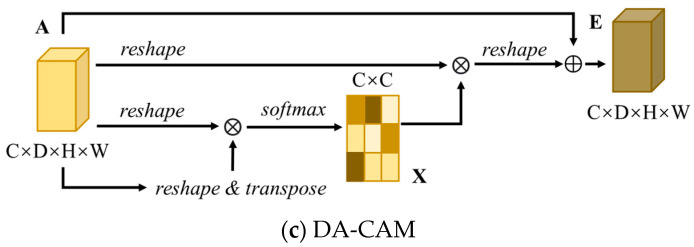
Structure of the DA-Block. The feature map undergoes one round of convolution and then is separately inputted into the DA-PAM and DA-CAM modules for feature extraction. The final output is obtained by combining different feature maps. The details of DA-PAM and DA-CAM are illustrated in (**b**,**c**).

The DA-Block consists primarily of two components: a Position Attention Module (DA-PAM) and a Channel Attention Module (DA-CAM). Figure 3 illustrates the specific structure of the DA-Block and the details of DA-PAM and DA-CAM are illustrated in Figure 3b,c.

#### 3.2.1. DA-PAM

DA-PAM [46,47] is responsible for capturing the spatial dependency relationship between any two positions in the feature map. Regardless of distance, similar features are correlated with each other, aiding in the extraction of effective spatial features. On the other hand, this module updates the features by weighted summation of all position features, where the weights are determined by the similarity of features between positions. The structural diagram of DA-PAM is illustrated in Figure 3b.

The specific procedure of DA-PAM is as follows:

(1) Input feature map *A*∈*R*^*C*×*D*^^×*H*×*W*^, where *C* represents the number of channels, *D* represents the depth, *H* represents the height, and *W* represents the width. Through three convolutional layers, three feature maps *P*, *Q*, and *M* are obtained, each with a size of *R*^*C*×*D*×*H*×*W*^.
(2) Let *N* = *D* × *H* × *W* denote the number of voxels. Reshape the feature maps *P*, *Q*, and *M* into *P*′, *Q*′, and *M*′, each with a size of *R*^*C*×*N*^.
(3) Perform transpose operation on feature map *P*′ to obtain *P*′∈*R*^*N*×*C*^, then multiply it with the feature map *Q*′. The result of matrix multiplication passes through a softmax layer to obtain the normalized weight map *S*∈*R*^*N*×*N*^, where *S* represents the spatial attention map, as shown in:
  (2) $$s_{ji}=\frac{\exp\left(P_{i}^{\prime }\cdot Q_{j}^{\prime }\right)}{\sum_{i=1}^{N}\exp\left(P_{i}^{\prime }\cdot Q_{j}^{\prime }\right)}$$
  where *s_ji_* denotes the influence of the *i*-th position on the *j*-th position. $P_{i}^{\prime }$ represents the *i*-th position of the transposed feature map *P*′, and $Q_{j}^{\prime }$ represents the *j*-th position of the transposed feature map *Q*′.
(4) Multiply the transposed weight map *S* with the feature map *M*′, and then scale it by a scale factor *α*. Reshape the result into *R*^*C*×*D*×*H*×*W*^. The initial value of *α* is set to 0, and it is iteratively trained to obtain larger weights.
(5) Finally, element-wise addition with the input feature map *A* yields the output *E*∈*R*^*C*×*D*×*H*×*W*^, as shown in:
  (3) $$E_{j}=\alpha \sum_{i=1}^{N}\left(s_{ji}\cdot M_{i}^{\prime }\right)+A_{j}$$

DA-PAM selectively aggregates spatial context, addressing the issue of intra-class inconsistency caused by convolution operations, effectively enhancing the spatial feature extraction capability.

#### 3.2.2. DA-CAM

Similar to DA-PAM, DA-CAM [46,47] focuses on the correlation between channels. DA-CAM is responsible for integrating relevant features between all channel mappings, adjusting the output weights of different channels, and selectively emphasizing channels that exhibit mutual dependencies, thereby enhancing feature representation capability. Figure 3c depicts the structural diagram of DA-CAM.

The specific procedure of DA-CAM is as follows:

(1) Let *N* = *D* × *H* × *W* denote the number of voxels. Reshape the input feature map *A* into *A*′∈*R*^*C*×*N*^.
(2) Multiply *A*′ with its transposed form *A*′∈*R*^*N*×*C*^, and the result passes through a softmax layer to obtain the normalized weight map *X*∈*R*^*C*×*C*^. *X* represents the channel attention map, as shown in:
  (4) $$x_{ji}=\frac{\exp\left(A_{i}^{\prime }\cdot A_{j}^{\prime }\right)}{\sum_{i=1}^{C}\exp\left(A_{i}^{\prime }\cdot A_{j}^{\prime }\right)}$$
(3) Multiply the transposed weight map *X* with the reshaped feature map *A*′, and then scale it by a scale factor *β*. Reshape the result into *R*^*C*×*D*×*H*×*W*^. The initial value of *β* is set to 0, and it is iteratively trained to obtain larger weights.
(4) Finally, element-wise addition with the input feature map *A* yields the output *E*∈*R*^*C*×*D*×*H*×*W*^, as shown in:
  (5) $$E_{j}=\beta \sum_{i=1}^{C}\left(x_{ji}\cdot A_{i}^{\prime }\right)+A_{j}$$

Compared to DA-PAM, DA-CAM does not involve the process of generating new feature maps through three convolutions, thus, maintaining the correlation between original channels. On the other hand, the final output features of DA-CAM are generated by the weighted sum of all channel features and the original features, contributing to the extraction of channel features.

#### 3.2.3. The Detailed Process of the DA-Block

The DA-Block integrates the DA-PAM and the DA-CAM, combining the advantages of position feature extraction and channel feature extraction. The DA-Block consists of two layers: the first layer introduces the DA-PAM, and the second layer introduces the DA-CAM.

Given the input feature map, in the first layer, the number of channels is reduced to one-sixteenth through a convolution operation, resulting in *x*_1_. This reduction in channels aids in feature extraction by DA-PAM. *x*_1_ is then inputted into DA-PAM for feature extraction, followed by another convolution, resulting in ${\overset{⌢}{x}}_{1}$, as described in:

(6) $$x_{1}=Conv(input)$$

(7) $${\overset{⌢}{x}}_{1}=Conv\left(PAM\left(x_{1}\right)\right)$$

The process is similar for the second layer, with DA-PAM replaced by DA-CAM, as shown in:

(8) $$x_{2}=Conv(input)$$

(9) $${\overset{⌢}{x}}_{2}=Conv\left(CAM\left(x_{2}\right)\right)$$

The obtained features ${\overset{⌢}{x}}_{1}$ and ${\overset{⌢}{x}}_{2}$ are aggregated and summed and, through another convolution, restored to the original number of channels, yielding the final output as shown in:

(10) $$output=Conv({\overset{⌢}{x}}_{1}+{\overset{⌢}{x}}_{2})$$

The DA-Block conducts feature extraction at both the positional and channel levels, aiming to enhance the depth of feature representation while preserving the intrinsic characteristics of the input mappings. In the encoder, convolutional layers, residual connections, and downsampling are combined to form consecutive convolutional modules. Each convolutional module halves the size of the input feature map while doubling the dimensions, maximizing feature expression while maintaining computational efficiency. The input image undergoes four consecutive convolutional blocks, gradually expanding the receptive field to capture important features. Subsequently, two DA-Blocks are applied to optimize the feature extraction process based on positional and channel-based attention mechanisms.

### 3.3. Convolutional Block Attention Module

**Figure 4:**
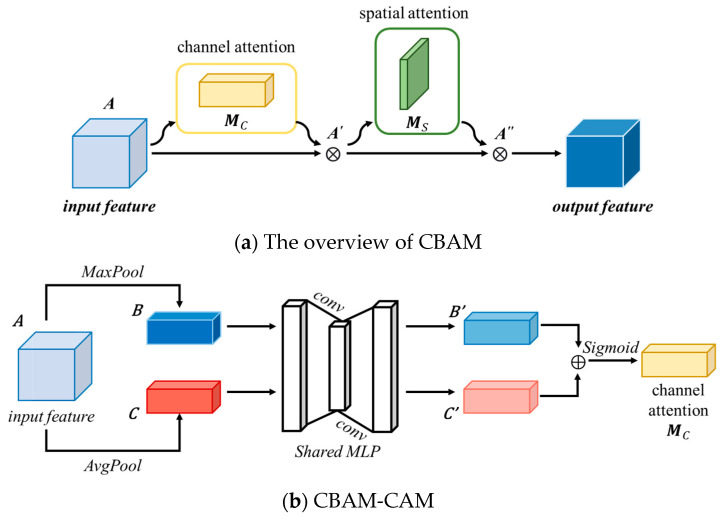

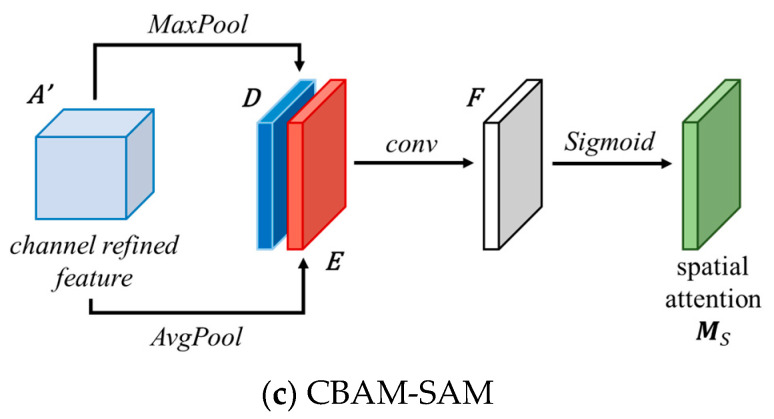
Structure of the CBAM. It realizes a sequential attention structure from channels to spatial dimensions. The details of CBAM-CAM and CBAM-SAM are illustrated in (**b**,**c**).

Traditional attention mechanisms based on convolutional neural networks tend to focus more on analyzing the channel domain, limited to considering the interaction between feature map channels. The Convolutional Block Attention Module (CBAM) [48] consists of both the Channel Attention Module (CBAM-CAM) and Spatial Attention Module (CBAM-SAM), realizing a sequential attention structure from channels to spatial dimensions. Spatial attention enables the neural network to focus more on pixel regions in the image that are crucial for classification while disregarding irrelevant regions. The channel attention is used to handle the allocation relationship among feature map channels. Simultaneously, attending to both dimensions enhances the attention mechanism’s effectiveness in improving model performance. The structure of CBAM is shown in Figure 4, and the details of CBAM-CAM and CBAM-SAM are illustrated in Figure 4b,c.

#### 3.3.1. CBAM-CAM

Compared to the DA-CAM in DA-Block, which generates channel attention maps by computing inter-channel similarities within feature maps, the CBAM-CAM utilizes global average pooling and global maximum pooling to capture global information from feature maps. Subsequently, convolutional layers processing and combining the results of these two pooling operations generate channel attention maps. The structure of the CBAM-CAM [48] is illustrated in Figure 4b. The specific process is as follows:

(1) Input feature map *A*∈*R*^*C*×*D*×*H*×*W*^, where *C* represents the number of channels, *D* represents the depth, *H* represents the height, and *W* represents the width. The feature map is separately subjected to max-pooling and average-pooling along the spatial dimensions, resulting in two different dimensional feature descriptions, *B* and *C*, both of size *R*^*C*×1×1×1^, as shown in:
  (11) $$B=MaxPool\left(A\right)=A_{\max}^{c}$$
  (12) $$C=AvgPool\left(A\right)=A_{avg}^{c}$$
  where $A_{\max}^{c}$ and $A_{avg}^{c}$ represent max-pooling and average-pooling, respectively.
(2) *B* and *C* are passed through a shared-weight Multilayer Perceptron (MLP) network, comprising two layers of convolution operations *W*_0_ and *W*_1_, resulting in new feature maps *B*′ and *C*′, as shown in:
  (13) $$B^{\prime }=MLP\left(B\right)=W_{1}\left(W_{0}\left(A_{\max}^{c}\right)\right)$$
  (14) $$C^{\prime }=MLP\left(C\right)=W_{1}\left(W_{0}\left(A_{avg}^{c}\right)\right)$$
(3) *B*′ and *C*′ are element-wise added and passed through a sigmoid activation function to obtain normalized channel attention weights *M_c_*(*A*), as shown in:
  (15) $$M_{c}\left(A\right)=\sigma \left(B^{\prime }+C^{\prime }\right)$$
  where *σ* represents the sigmoid activation function.

This process describes the operation of the channel attention mechanism in CBAM, providing a mechanism for the network to dynamically adjust channel-wise feature representations.

#### 3.3.2. CBAM-SAM

The structure of the CBAM-SAM [48] is illustrated in Figure 4c. This module processes the output feature maps from the channel attention module in the spatial domain. The specific process is outlined as follows:

(1) Input feature map *A*′∈*R*^*C*×*D*×*H*×*W*^. Max-pooling and average-pooling are separately applied along the channel dimension of the feature map, resulting in two different dimensional feature descriptions, *D* and *E*, both of size *R*^1×*D*×*H*×*W*^, as shown in:
  (16) $$D=MaxPool\left(A^{\prime }\right)={A^{\prime }}_{\max}^{s}$$
  (17) $$E=AvgPool\left(A^{\prime }\right)={A^{\prime }}_{avg}^{s}$$
  where ${A^{\prime }}_{\max}^{c}$ and ${A^{\prime }}_{avg}^{s}$ represent max-pooling and average-pooling, respectively.
(2) *D* and *E* are stacked along the channel dimension and passed through a convolution operation followed by a sigmoid activation function to obtain normalized spatial attention weights *Ms*(*A*′), as shown in:
  (18) $$M_{s}\left(A^{\prime }\right)=\sigma \left(W\left(D;E\right)\right)=\sigma \left(W\left({A^{\prime }}_{\max}^{s};{A^{\prime }}_{avg}^{s}\right)\right)$$
  where *W* represents the convolution operation.

This process describes the operation of the spatial attention mechanism in CBAM, enabling the network to dynamically adjust spatial feature representations according to their importance for classification.

#### 3.3.3. The Detailed Process of the CBAM

CBAM consists of two parts: the CBAM-CAM and the CBAM-SAM. The input feature map *A* undergoes processing by the channel attention module *M_c_*, resulting in channel attention weights. These weights are then multiplied element-wise with the input feature map and fed into the spatial attention module *Ms*, where spatial normalization weights are obtained. Finally, the output of the spatial attention module is multiplied element-wise with its input feature map to yield the final feature map. The computational process is described by:

(19) $$A^{\prime }=M_{c}\left(A\right)⊗A$$

(20) $$A^{\prime \prime }=M_{s}\left(A^{\prime }\right)⊗A^{\prime }$$

where ⨂ represents element-wise multiplication. *A*′ and *A*″ represent the output feature maps after passing through the two modules, respectively.

## 4. Experiments

### 4.1. Datasets and Evaluation

To evaluate the proposed method, we conducted experiments on the LiTS dataset [49], which is dedicated to liver and tumor segmentation in CT scans. This dataset comprises data from seven different medical centers, consisting of 131 training cases and 70 testing cases. CT scans are accompanied by reference annotations made by trained radiologists for the liver and tumors. The dataset contains 908 lesions. There are significant variations in image quality, spatial resolution, and visual appearance within the dataset. The in-plane resolution ranges from 0.6 mm × 0.6 mm to 1.0 mm × 1.0 mm, and the slice thickness (inter-slice spacing) ranges from 0.45 mm to 6.0 mm. All scans have a fixed axial slice size of 512 × 512 pixels, but the number of slices per scan varies from 42 to 1026. Data augmentation techniques, such as random cropping, scaling, and rotation, were applied to all data to enhance training accuracy.

Four image segmentation evaluation metrics were utilized in this study, including the Dice Coefficient (DSC), Volumetric Overlap Error (VOE), Hausdorff Distance (HD), and Root Mean Square Symmetric Surface Distance (RMSD). These metrics were employed to assess the accuracy, precision, and robustness of the models.

(1) The *DSC* is a measure of similarity often used to compute the similarity or overlap between the predicted segmentation result *V_seg_* and ground truth *V_gt_*, particularly effective in cases where object boundaries are not clearly defined. Its formula is given by:
  (21) $$DSC=2\frac{V_{seg}\cap V_{gt}}{V_{seg}+V_{gt}}$$
(2) The *VOE* is employed to quantify the overlap between two volumes. It is calculated as follows:
  (22) $$VOE=1-\frac{V_{seg}\cap V_{gt}}{V_{seg}\cup V_{gt}}$$
(3) The *HD* is a metric used to measure the similarity between sets of points. Given two point sets *A* and *B*, it computes the maximum distance from each point in *A* to the nearest point in *B*. The formula for *HD* is:
  (23) $$HD\left(A,B\right)=\max\left(\underset{a\in A}{\sup}\;\underset{b\in B}{\inf}\;d\left(a,b\right),\underset{b\in B}{\sup}\;\underset{a\in A}{\inf}\;d\left(a,b\right)\right)$$
  where *d*(*a*, *b*) represents the distance between points *a* and *b*. The Hausdorff 95 (HD95) is evaluated by sorting the distances in ascending order and taking the 95th percentile value. This experiment was evaluated using *HD95*.
(4) The *RMSD* is utilized to quantify the difference between structures. Its formula is given by:
  (24) $$RMSD=\sqrt{\frac{1}{n}\sum_{i=1}^{n}{\left(x_{i}-y_{i}\right)}^{2}}$$
  where *x_i_* and *y_i_* are the coordinates of corresponding voxels in the structures, and *n* is the number of voxels.

### 4.2. Implementation Details

All experiments were conducted using the PyTorch 2.0.0 framework and were run on a single NVIDIA RTX 3080 GPU.

In the image preprocessing stage, we first unified the Hounsfield intensity to [−200, 200] to remove irrelevant organs. Then, we reduced the resolution of the image from 512 × 512 to 256 × 256. Finally, the spacing of the slice axis was set to 1 mm.

**Figure 5:**
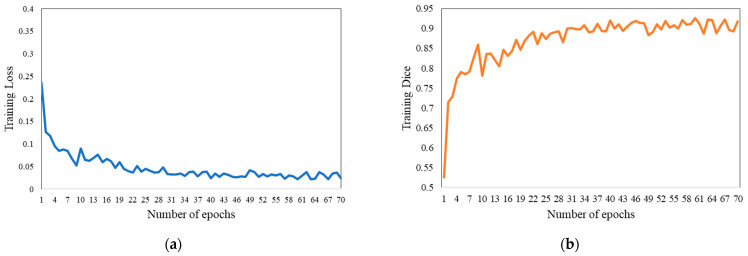
Training diagrams of our approach. (**a**,**b**), respectively, depict the training loss and Dice coefficient curves.

In the training process, we set the epoch to 200, the early-stopping to 30, and the batch size to 1. We used standard Adam to optimize the objective function, configured with a learning rate of 1 × 10^−3^. The loss function is Tversky loss [50], defined as:

(25) $$T\left(V_{seg},V_{gt}\right)=\frac{\left|V_{seg}\cap V_{gt}\right|}{\left|V_{seg}\cap V_{gt}\right|+\alpha \left|V_{seg}-V_{gt}\right|+\beta \left|V_{gt}-V_{seg}\right|}$$

where *α* is 0.7 and *β* is 0.3. Figure 5a,b, respectively, depict the training loss and Dice coefficient curves of our approach.

### 4.3. Segmentation Results

#### 4.3.1. Ablation Studies

To validate the effectiveness of the proposed method, we conducted two sets of ablation experiments:

(1) Effect of the CBAM in Encoder and Skip Connection.

In this study, we evaluated the impact of integrating the CBAM module into the encoder and skip connections on the segmentation performance of the model. Table 1 presents the results of this study. Specifically, CBAM was introduced into the encoder and each skip connection. The results demonstrated a noteworthy improvement: the *DSC* of the liver increased from 91.72% to 92.47%, and the *HD95* index decreased from 30.54 mm to 29.03 mm. This indicates that adding CBAM modules to the encoder and each skip connection provides finer features to the decoder and reduces feature loss during upsampling, thereby reducing the risk of overfitting and enhancing model stability. Additionally, we also experimented with integrating CBAM into each of the three skip connections separately to determine the optimal architectural layout for improving model performance. Specifically, when CBAM was added only to the first skip connection, the *DSC* decreased from 91.72% to 90.87%, and similar decreasing trends were observed when CBAM was added to the second and third skip connections. However, significant improvements were observed when CBAM was integrated into all layers, with *DSC* reaching 92.47% and *HD95* decreasing to 29.03 mm. In summary, according to the results in Table 1, integrating CBAM into both encoder and skip connections effectively enhances the medical Image segmentation capabilities.

(2) Effect of the DA-Block in Encoder.

In this study, we evaluated the impact of integrating the DA-Block into the encoder on the segmentation performance of the model. Table 2 lists the results of this study. The results demonstrated an increase in the DSC of the liver from 91.72% to 92.25% and a decrease in the HD95 from 30.54 mm to 29.72 mm. These findings suggest that integrating the DA-Block into the encoder, introducing position attention and channel attention mechanisms effectively improves the accuracy of medical image segmentation.

**Figure 6:**
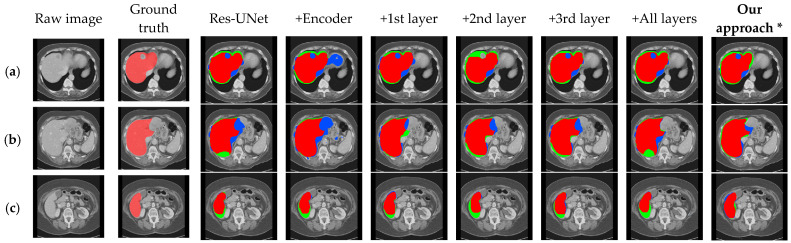
Qualitative segmentation results on the ablation models for the effect of the CBAM in encoder and skip connection. (**a**–**c**), respectively, show the segmentation results under different cross-sections. (* Our approach in this experiment is without DA-Block).

**Figure 7:**
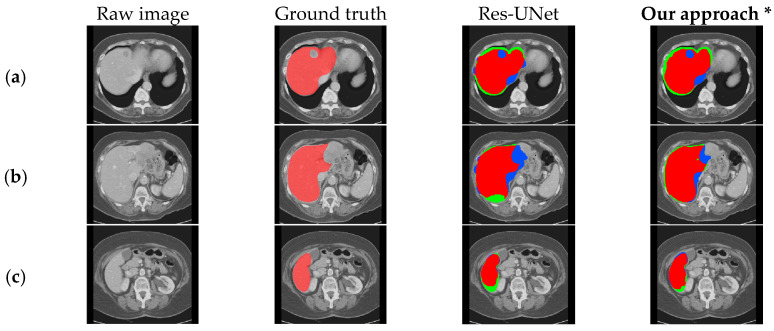
Qualitative segmentation results on the ablation models for the effect of the DA-Block in Encoder. (**a**–**c**), respectively, show the segmentation results under different cross-sections. (* Our approach in this experiment is without CBAM).

Visual results corresponding to the above two sets of ablation experiments are shown in Figure 6 and Figure 7. In these images, the light red labels in the second column represent the ground truth. In the remaining columns, the dark red areas indicate the overlap between the segmentation results and the ground truth, the blue areas represent the regions where the segmentation results exceed the ground truth, and the green areas denote the regions where the segmentation results are smaller than the ground truth.

#### 4.3.2. Comparison with Other Methods

**Figure 8:**
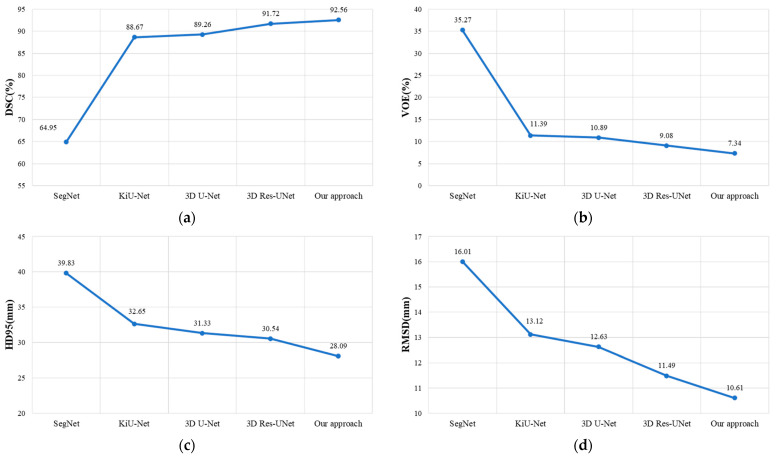
The experimental results of our approach compared with other models on the LiTS dataset. (**a**–**d**), respectively, presents the comparisons of our approach with other models in terms of DSC, VOE, HD95, and RMSD.

We compared our method with 3D U-Net [37], Res-UNet, SegNet [51], and KiU-Net [52], and the experimental results are shown in Table 3 and Figure 8. To validate the effectiveness of our method, all experiments were conducted on the LiTS dataset using the same preprocessing methods.

It can be observed that our method achieved DSC, VOE, HD95, and RMSD evaluation metric results of 92.56%, 7.34%, 28.09 mm, and 10.61 mm, respectively. Compared to Res-UNet, our method demonstrated an improvement of 0.84% in DSC, a reduction of 1.74% in VOE, a decrease of 2.45 mm in HD95, and a decrease of 0.88 mm in RMSD. This indicates that our method outperforms Res-UNet in liver segmentation and organ edge prediction.

**Figure 9:**
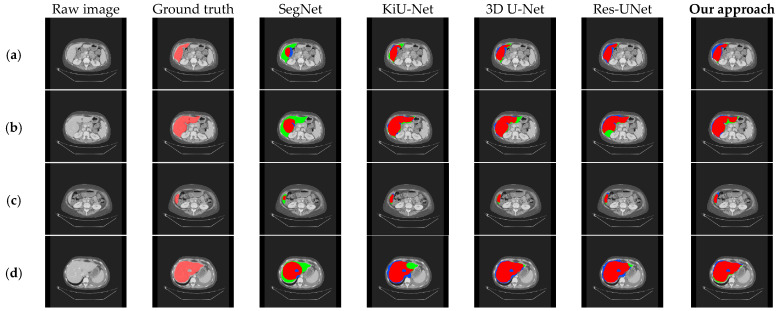
Comparison of qualitative results between our approach and existing models on the LiTS dataset. (**a**–**d**), respectively, show the segmentation results under different cross-sections.

To further confirm that our model exhibits better segmentation performance compared to other methods, we visualized the segmentation results of our method and those of 3D U-Net, Res-UNet, SegNet, and KiU-Net, as shown in Figure 9. Compared with other models, it can be observed that our method performs better in liver edge segmentation, capturing more details of the liver edge. Thus, our method demonstrates superior segmentation quality and visualization performance compared to other methods. However, for the blank areas inside the liver (i.e., tumor regions), none of the models can accurately segment, indicating the need for further optimization in future work.

## 5. Conclusions

To address the limitations of existing methods, such as weak contextual analysis ability and semantic information loss in liver CT image segmentation, we propose a novel dual-attention 3D U-Net liver segmentation algorithm based on CT images, which integrates DA-Block and CBAM with 3D Res-UNet. The innovation of this method lies in the following aspects: (1) Introducing residual connections allows the network to better capture multi-scale information, alleviate semantic information loss, and enhance image segmentation performance. (2) Proposing the encoder structure of DA-Block focuses on specific image positions and channel features to improve model performance. (3) By introducing CBAM in skip connections, the semantic gap between the encoder and decoder is effectively bridged, optimizing feature maps and further improving liver segmentation performance. Experimental results demonstrate a significant performance improvement of our proposed model compared to Res-UNet on the LiTS dataset.

This study validates the effectiveness of the proposed method for liver segmentation and demonstrates its potential for extension to other organ segmentation tasks. In future research, we will conduct experiments and validation on a multi-organ abdominal dataset. Additionally, we will focus on optimizing the decoder part of the architecture and explore research avenues such as reducing the computational complexity of DA-Block without compromising model performance.

## Figures and Tables

**Table 1:** Effects of combinatorial placement of CBAM and DA-Block in the encoder and through Skip connections on performance metrics. The ↑ for DSC indicates better segmentation with higher values, while the ↓ for HD95 indicates better segmentation with lower values.

Methods *	Encoder with CBAM	Skip with CBAM			DSC↑	HD95↓
		1st Layer	2nd Layer	3rd Layer		
Res-UNet					91.72	30.54
Our approach	√				90.63	34.21
Our approach		√			90.87	30.67
Our approach			√		91.61	28.74
Our approach				√	90.15	30.99
Our approach		√	√	√	91.33	**28.81**
Our approach	√	√	√	√	**92.47**	29.03

* The methods in this experiment are all without DA-Block.

**Table 2:** Effects of DA-Block in the encoder on performance metrics.

Methods	Encoder with DA-Block	DSC↑	HD95↓
Res-UNet		91.72	30.54
Our approach (without CBAM)	√	**92.25**	**29.72**

**Table 3:** Experimental results on the LiTS dataset.

Methods *	DSC (%)	VOE (%)	HD95 (mm)	RMSD (mm)
SegNet	64.95	35.27	39.83	16.01
KiU-Net	88.67	11.39	32.65	13.12
3D U-Net	89.26	10.89	31.33	12.63
3D Res-UNet	91.72	9.08	30.54	11.49
**Our approach**	**92.56**	**7.34**	**28.09**	**10.61**

* All networks in this research are 3D networks.

## Data Availability

The data presented in this study are available on request from the corresponding author.
